# Microstamped Petri Dishes for Scanning Electrochemical Microscopy Analysis of Arrays of Microtissues

**DOI:** 10.1371/journal.pone.0093618

**Published:** 2014-04-01

**Authors:** Adithya Sridhar, Hans L. de Boer, Albert van den Berg, Séverine Le Gac

**Affiliations:** BIOS Lab on a Chip Group, MESA+ Institute of Nanotechnology, University of Twente, Enschede, The Netherlands; LAAS-CNRS, France

## Abstract

While scanning electrochemical microscopy (SECM) is a powerful technique for non-invasive analysis of cells, SECM-based assays remain scarce and have been mainly limited so far to single cells, which is mostly due to the absence of suitable platform for experimentation on 3D cellular aggregates or microtissues. Here, we report stamping of a Petri dish with a microwell array for large-scale production of microtissues followed by their *in situ* analysis using SECM. The platform is realized by hot embossing arrays of microwells (200 μm depth; 400 μm diameter) in commercially available Petri dishes, using a PDMS stamp. Microtissues form spontaneously in the microwells, which is demonstrated here using various cell lines (*e.g*., HeLa, C2C12, HepG2 and MCF-7). Next, the respiratory activity of live HeLa microtissues is assessed by monitoring the oxygen reduction current in constant height mode and at various distances above the platform surface. Typically, at a 40 μm distance from the microtissue, a 30% decrease in the oxygen reduction current is measured, while above 250 μm, no influence of the presence of the microtissues is detected. After exposure to a model drug (50% ethanol), no such changes in oxygen concentration are found at any height in solution, which reflects that microtissues are not viable anymore. This is furthermore confirmed using conventional live/dead fluorescent stains. This live/dead assay demonstrates the capability of the proposed approach combining SECM and microtissue arrays formed in a stamped Petri dish for conducting cellular assays in a non-invasive way on 3D cellular models.

## Introduction

Scanning electrochemistry microscopy (SECM) is a powerful technique to map the topography and activity of biological systems with a micrometer-scale resolution [1], [2]. SECM analysis of biological samples does not require extensive sample preparation times unlike traditional fluorescence and colorimetric assays [3]. More significantly fluorescence assays are highly invasive, and can have adverse effects on cell health due to labelling and strong illumination required for measurements [4], [5]. On the contrary, SECM measurements are non-invasive and do not suffer from issues such as photobleaching. This capability is particularly interesting for continuous monitoring of samples, which is precluded in many traditional assays requiring fixation and permeabilization. A comprehensive overview of the advances in SECM-based imaging of living systems has recently been published by Bergner et al [6]. A large part of the reported work focused on oxygen measurements to assess the cell respiratory activity, as a marker for their viability or status [7], [8]. Interestingly, SECM can detect more rapidly changes in cellular activity than conventional live/dead stains [9]. Besides, intracellular measurements of the cell redox activity [10], [11] were reported as well as assays to evaluate the differentiation status of embryonic stem cells using an indirect enzymatic assay [12], [13].

Although these assays demonstrate the powerful capabilities of a SECM approach for biological measurements, they are mostly limited to single cell or monolayer models, which raises two essential issues. First, these assays fail to exploit the essence of SECM as a scanning probe technique to analyse large surface areas. Second, single cells and monolayers are not biologically relevant models, especially for studying complex biological processes [14]. Altogether, a more appropriate approach would consist of performing SECM measurements on arrays of 3D cell aggregates such as spheroids, which are acknowledged as closer models to the *in vivo* situation [15].

We hypothesize, the reason why the majority of SECM assays are preferentially performed on simple cellular models is the same as why monolayer models remain ubiquitous in cell biological research: a lack of suitable platforms, on one hand, compatible with laboratory equipment such as SECM, and, on the other hand, suitable for large scale production of homogenously-sized microtissues [16]. To solve the latter issue, the use of microwell arrays has been proposed, and previous work in our group employed microwell arrays made from “academic” materials such as polydimethylsiloxane (PDMS) [17] and agarose [18]. However, these soft materials are difficult to integrate with automated systems [19] and normally, they are processed as thick layers to improve handling. Furthermore, both agarose and PDMS are porous materials, in which oxygen can permeate. These two aspects preclude their use for SECM measurements, where the approach of the tip to the surface is monitored using both an inverted microscope and negative feedback [2]. In this context, rigid and non-porous materials such as thermoplastics are better candidates to realize microwell arrays and platforms for SECM measurements on microtissues [19], [20]. Polystyrene (PS) for instance has been widely used in biology for decades and thoroughly characterized for cell-based assays, is a good candidate material to realize such platforms.

Additionally, microwell arrays are advantageous for SECM measurements on cells and tissues as cross-talk between samples is avoided by spacing apart wells with sufficient and well-defined distance. In that context, a Si/PDMS hybrid device has been reported for viability assay on spheroids [21], PDMS microwells have been developed for sensitive analysis of enzymatic activity of yeast cells [22], and more recently, thin-film microwells have been developed for assessing multi drug resistance on monolayer cultures [11]. However, these devices not only suffer from material limitations discussed previously but also cannot be used universally across different equipment unlike the commercially available PS based culture ware.

**Figure 1:**
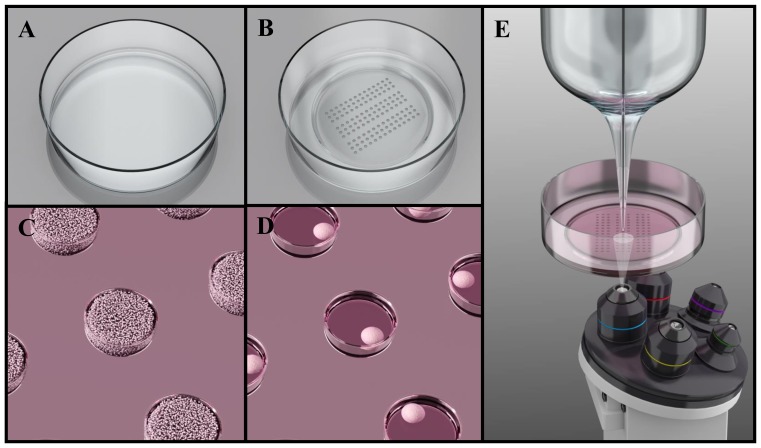
Platform for SECM-based microtissues assays following *in situ* microtissue production and integrated in a Petri dish. Schematic representations of a commercially available Petri dish before (A) and after (B) microstamping of a microwell array using hot embossing. The stamped Petri dishes are subsequently employed for tissue formation: (C) cells are seeded with a high concentration in the stamped Petri dishes (>500,000 cells/Petri dish), which are subsequently centrifuged to force cells to accumulate in the microwells. After ca. 24 h, cells have self-aggregated into microtissues in the microwells (D). (E) The platform is suitable for SECM measurements, as well as with other standard imaging techniques such as conventional microscopy. Images not to scale.

Therefore, we propose in this paper a simple and user-friendly platform for SECM-based measurements on microtissues after their *in situ* formation, which is realized in commercially available Petri dishes (Fig. 1). Specifically, a microwell array is stamped in a Petri dish using hot embossing technique. Next, we report spontaneous microtissue formation in the resulting platform, after surface coating and cell seeding. Microtissues produced from various cell lines (C2C12, MCF-7, HepG2 and HeLa) are subsequently characterized for their size and cell organization. Finally, we demonstrate the suitability of the platform for SECM measurements and *in situ* experimentation on microtissues. For that purpose, we perform a standard live/dead assay on HeLa microtissues, which are analysed before and after exposure to a model drug, using scanning electrochemical microscopy by monitoring oxygen consumption along with standard fluorescence microscopy.

## Experimental Section

### Fabrication of PDMS stamps

Master molds for the preparation of the PDMS stamps are fabricated in the clean rooms of the MESA+ Institute for Nanotechnology using standard photolithography techniques. They consist of 200 μm high SU-8 patterns on a silicon (Si) wafer. A hydrophobic coating of FDTS (perfluorodecyltrichlorosilane; ABCR, GmBH, Germany) is applied to this SU-8/Si master via molecular vapour deposition. This coating is notably essential for the durability of the SU-8 master and to facilitate the release of the PDMS stamps [23]. PDMS stamps are produced using standard soft lithography, as extensively described in the literature [24]. In brief, a two component kit (Sylgard 184, Dow Corning, Farnell, Maarssen, The Netherlands) consisting of pre-polymer and curing agent is employed, these two components being mixed in a 10∶1 weight ratio. This mixture is subsequently degassed, poured onto the SU-8/Si mold, and degassed again to remove any air bubble. Curing is achieved at 60°C overnight. After curing, the PDMS stamps are released from the master mould, and cut into individual chips.

### Hot embossing of PS Petri dish

CELLSTAR cell culture dishes (35 mm diameter; Greiner Bio-One GmbH, Alphen aan den Rijn, The Netherlands), are patterned using PDMS stamps for hot embossing. Prior to hot embossing, PDMS stamps are cleaned for 10 min in isopropyl alcohol (IPA; Sigma-Aldrich, Zwijndrecht, The Netherlands) in an ultrasonic bath, and subsequently dried for 15 min at 60°C. Microwell arrays are hot embossed using a custom setup (Fig. S1 in File S1) for local reshaping of the Petri dish as thoroughly discussed in the results section. Hot embossing proceeds in 3 steps: First, the setup is first pre-heated using a soldering station (Tooltronics, The Netherlands) to the desired temperature (180°C) and the applied temperature is monitored using an internal thermocouple. Subsequently, the dish is placed together with the PDMS stamp in the set-up, and pressure is applied with the help of a screw for 10 min. The applied pressure is measured using a model 1022 load cell (Farnell, The Netherlands). Finally, the set-up is cooled down for 15 min before its disassembly and release of the stamped Petri dish.

### Cell culture

Mouse myoblasts (C2C12), human cervical cancer (HeLa), human liver carcinoma (HepG2), and human breast adenocarcinoma (MCF-7) cell lines (all cells from ATCC, Germany) are cultured in Dulbecco's Modified Eagle Medium (DMEM) with 10% v/v foetal calf serum, 2% v/v Penicillin/Streptomycin and 1% v/v L-glutamine (all culture reagents from Gibco, Invitrogen, Breda, The Netherlands). Cells are cultured under standard conditions at 37°C, 5% CO_2_ in a humidified incubator. C2C12 cells are not allowed to form confluent monolayers and all cell types are passaged every 3 days. Cells grown in monolayer are detached to form cell suspensions using Trypsin (2 min incubation in a 0.25% Trypsin-EDTA solution (Gibco, Invitrogen)), followed by centrifugation of dissociated cells at 1500 RPM for 5 min. Cells are re-suspended in growth medium and the cell density is determined using a haemocytometer.

### Spheroid formation and culture

A cell-repellent coating based on Pluronic F-127 (BASF, USA) is applied to the microstamped Petri dishes to promote the formation of cell aggregates. Before application of the coating, the Petri dishes are cleaned for 10 min in IPA in an ultra-sonic bath and dried for 15 min at 60°C. A 1% w/v (1 g in 100 mL) Pluronic solution is prepared in DI water, and 3 mL of this solution is added to each Petri dish. The dishes are then centrifuged at 3000 RPM for 5 min to remove air bubbles, and incubated at 37°C overnight with the Pluronic solution. Thereafter, the Petri dishes are washed once with PBS. A 500 μL cell suspension with 500,000 cells in culture medium is seeded in the Petri dish, followed by 2 mL of culture medium. The dish is then centrifuged at 3000 RPM for 5 min. After centrifugation, the culture medium is changed to remove the excess of cells present outside the microwells. Subsequently, 2 mL of fresh medium is added to the Petri dish, which is placed back in the incubator. The medium is replaced after 24 h, and subsequently every 48 h. To measure the tissue sizes, images of microtissue array for each cell type (3 Petri dishes per cell type) are captured using optical microscopy (IX51 Olympus microscope). The tissue diameters are subsequently measured using Image processing software (ImageJ, NIH, USA).

### F-actin staining and Hoechst nuclear staining

Tissues are pipetted out of the microwells and washed twice with PBS. Following this, they are fixed using 4% paraformaldehyde (PFA; Sigma-Aldrich) at 4°C for 30 min. Thereafter, tissues are permeabilized with 0.1% triton X-100 (Sigma-Aldrich) for 10 min at 37°C. For actin staining, tissues are incubated with a PBS solution containing 1% w/v BSA (Sigma-Aldrich) and 10 μg/mL fluorescein phalloidin (Invitrogen) at 37°C for 2 h. Following this, the cell nucleus is stained with a 1 μg/mL Hoechst 33342 (Invitrogen) in PBS with 1% w/v (1 g in 100 mL) BSA (incubation at 37°C for 30 min). The samples are washed twice with PBS between every step. Tissues are finally imaged in PBS using confocal laser scanning microscopy (Nikon A1R-A1 confocal system; 40x oil immersion objective) in the BioNanoLab of the MESA+ Institute for Nanotechnology.

### Scanning Electrochemical Microscopy to assess microtissue respiratory activity

SECM measurements are performed in a three electrode configuration using an electrochemical probe scanner (ElProScan, HEKA, Germany) in feedback mode [2]. A Pt/Hg electrode is used as the working electrode with Ag wire reference and Pt wire counter electrode. Mercury is deposited on the 10 μm Pt working electrode by electrochemical deposition to improve its sensitivity and stability for oxygen measurements [25]. All biological measurements are carried out in HEPES buffer (10 mM HEPES, 150 mM NaCl, 4.2 mM KCl, 2.7 mM MgCl_2_, 1 mM NaH_2_PO_4_ and 11.2 mM D-(+)-Glucose; all reagents from Sigma-Aldrich) at 35°C. Microtissues, are analysed using SECM by scanning along lines above the tissues with a 10 μm/s rate, at different heights above the microtissues, while ensuring the electrode passes above the center of the microtissue.

### Live/dead assay and measurements using membrane integrity markers and fluorescence microscopy

A live/dead assay is performed using a combination of standard viability stains, calcein AM (acetoxymethyl) and propidium iodide (PI, both Invitrogen). First, live microtissues are stained using calcein AM. Before staining, the medium is carefully drained from the Petri dish and tissue samples are washed twice with PBS. Culture medium containing 1 μg/mL of the calcein AM is added to the Petri dish. After 1 h incubation at 37°C, the medium is removed and the sample washed twice with PBS. To induce cell death, microtissues are exposed to a 50% ethanol solution in culture medium for 45 min. After washing twice with PBS, culture medium supplemented with 1 μg/mL PI is added to the Petri dish. After 1 h incubation at 37°C and washing with PBS twice, the sample is imaged using fluorescence microscopy (IX51 Olympus inverted microscope equipped with a X-cite 120 PC Hg lamp using a 2x objective).

## Results

### Platform fabrication

**Figure 2:**
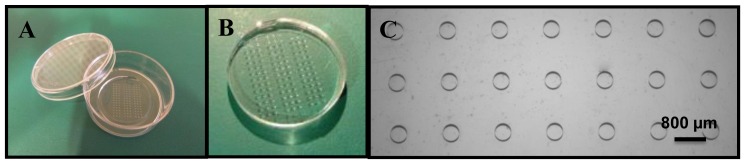
Fabrication of the stamped Petri dish. PDMS mold (A) employed for stamping microwell arrays in polystyrene-based Petri dish by hot embossing and presenting three arrays of 42 pits (200 μm height; 400 μm diameter, and 800 μm spacing). (B). Picture of a Petri dish after hot embossing of the microwell array. (C) Microscopy picture showing 21 independent wells in the polystyrene substrate.

The platform developed for analysis of tissues using SECM consists of a microwell array stamped in a commercially available Petri dish. For stamping microwell arrays in a Petri dish while preserving the overall shape of the dish, a dedicated set-up is developed (Fig. S1 in File S1). Specifically, the heat and pressure required for hot embossing are applied locally for selective re-shaping of the PS substrate in the center of the Petri dish. The Petri dish is placed in a Teflon cup that contains a brass disc in its center. This alloy disc which acts as heating element, is connected to a solder iron. Furthermore, its diameter matches that of the area to be stamped, *i.e*., of the resulting microwell array. The PDMS stamp, employed for hot embossing [26], is placed in a dedicated part which is screwed down onto the Petri dish, and the applied pressure is adjusted using the screw while the Brass disc is heated. The applied force for hot embossing measured using a load cell is in the order of 20 to 30 N (c.a. 0.5 MPa), which is in agreement with the forces reported in literature for soft embossing using PDMS molds [27]. In the current configuration, this parameter is not controlled with high precision, but the applied pressure proved to be roughly reproducible from experiment to experiment. Fig. 2 presents pictures of a PDMS master employed for hot embossing and of a stamped Petri dish, together with a microscopic picture of an array of microwells stamped in PS.

Using this set-up, microwell arrays are successfully realized in the bottom part of dishes while maintaining their overall shape. For hot embossing, the temperature applied must be above the glass transition temperature of polystyrene (95°C) [20], [28], and optimization of the fabrication parameters shows that an applied temperature of 180°C yields the best fabrication results. Using lower temperatures (<170°C), the circular microwell shape is not replicated uniformly while higher temperatures (>190°C) results in heating of a larger surface area, which starts to affect the Petri dish shape. Fabrication at optimized settings (180°C, 10 min heating and 15 min cooling) gives homogenous microwell arrays, the dimensions of the PDMS master (400 μm diameter, 200 μm depth and 800 μm spacing between wells) being replicated with good fidelity in the polystyrene (depth 184±2 μm and diameter 395±3 μm, n = 30; 3 devices and 10 microwells per device), as thoroughly characterized using profilometry techniques (Fig. S2 in File S1).

In this paper, we stamped relatively large structures which are arrayed as 3 series of 3 lines of 14 tissues (Fig. 1B & Fig. 2C). However, by changing the density and the size of the microwells, thousands of microtissues can be included per platform, and the dimensions of the microtissues varied. Using the same approach, we have also produced Petri dishes with arrays of >100,000 microwells (25 μm diameter and 20 μm depth) for capture of individual cells (Fig. S3 in File S1). Furthermore, a variety of techniques are available for processing of thermoplastics, such as injection moulding [29], soft lithography [30], and hot embossing [20]. In this work, hot embossing is utilized since it lends itself well to local reshaping of the material while keeping the initial overall shape of the dish, which is essential here to preserve the Petri dish format.

### Microtissue production and characterization

**Figure 3:**
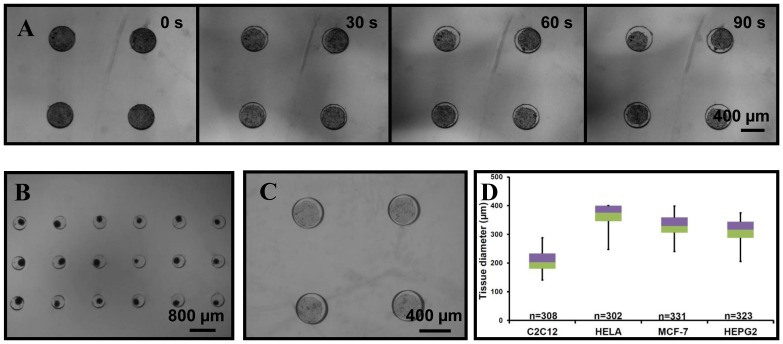
Microtissue formation in the stamped Petri dish. (A) Pictures extracted from the movie presented in supplementary materials and showing the formation of C2C12 microtissues. (B) C2C12 spheroids array 24 h after cell seeding in a stamped Petri dish; enlarged view on 18 microtissues. (C) HepG2 spheroids in a stamped Petri dish after 24 h of culture; enlarged view on 4 microtissues. (D) Box-plot of the microtissue size 24 h after seeding showing that all four cell lines form homogenous microtissues (HeLa 369±33 μm; MCF-7 331±35 μm; Hep G2; 311±35 μm; and C2C12 206±34 μm).

**Figure 4:**
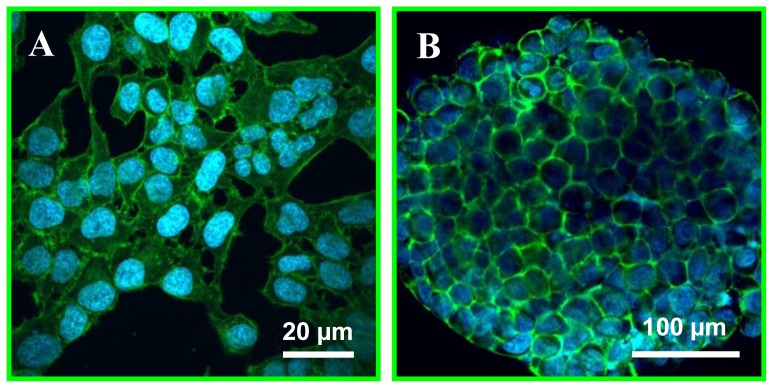
Actin organization in microtissues. Off-Petri dish confocal imaging of HeLa monolayers (A) and microtissues (B) after staining of their nucleus with Hoechst (blue) and actin (green) using FITC-phalloidin showing complete reorganisation of the cell matrix protein (actin).

Next, microtissues are successfully produced from various cell lines (HeLa, MCF-7, HepG2 and C2C12) in the stamped Petri dish. Before cell seeding, a 1% w/v (1 g in 100 mL) Pluronic F-127 coating is applied on the dish. Pluronic is a non-ionic surfactant polyol which forms a hydrophilic layer on the PS hydrophobic surface. This coating enables to eliminate cell-substrate interactions by preventing protein adhesion on the PS surface [31]. As a result, it forces cell-cell interactions leading to self-assembling microtissues in each microwell (Fig. 3A and File S2). After ca. 24 h, microtissues reach a “stable” diameter and stop compacting. Following this, cell proliferation is observed (data not shown) for most of the employed cell lines, which results in an increase of the tissue size. After 24 h, the tissue diameter is determined for all four cell lines, and Fig. 3 B & C show pictures of microtissues prepared from HeLa and HepG2 cells after 24 h of culture in the platform. All form homogenously-sized microtissues (ca. 10% deviation), whose diameter is dependent on the cell type. For example, smooth muscle cells (C2C12; 206±34 μm) contract more than liver (HepG2; 311±35 μm) and breast carcinoma (MCF-7; 331±36 μm) cells for a fixed cell seeding density in the microwell array (Fig. 3 B & D). Following this, the tissue organization is characterized by imaging the distribution of matrix proteins such as actin. For that purpose, microtissues are stained with phalloidin coupled to a fluorophore (fluorescein), and imaged with confocal microscopy. As shown on Fig. 4B, actin is solely found at the cell junctions in HeLa microtissues as expected, while in monolayers (Fig. 4A), this matrix protein is uniformly spread across the cells.

### SECM-based analysis of microtissues: Oxygen consumption by live microtissues

**Figure 5:**
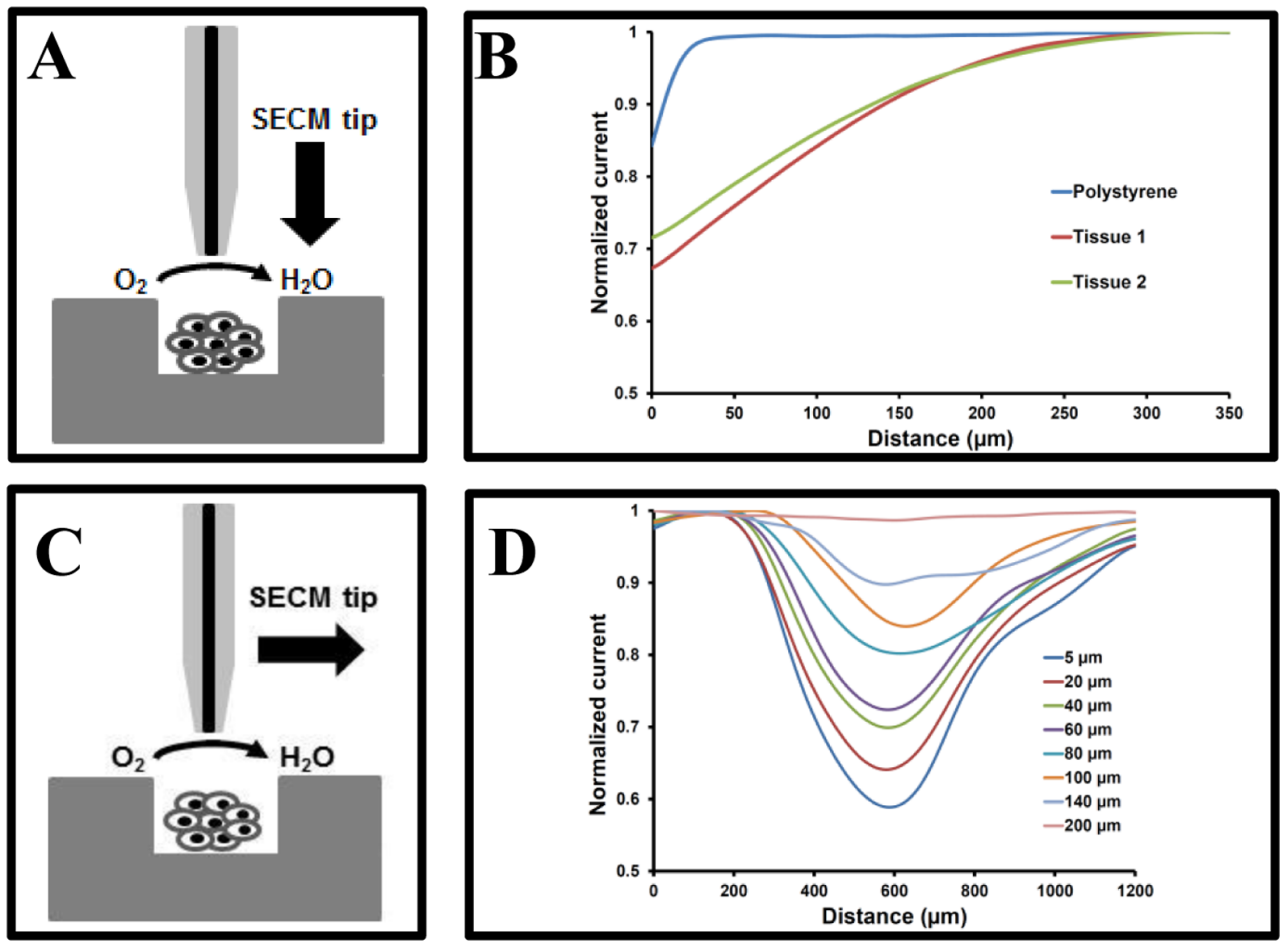
SECM assessment of the microtissue respiratory activity. (A) and (B) variations of the oxygen reduction current in solution as a function of the microelectrode surface distance when the microelectrode is approached from the bulk towards the Petri dish or the microtissue surface (center of the microtissue). (C) and (D) SECM scans over a 2-day old HeLa microtissue at different heights, along a line above the center of the microtissues, to monitor the microtissue oxygen consumption (Hg-coated Pt microelectrodel −0.6 V vs. Ag wire (reference electrode); scan rate of 10 μm/s). Currents are normalized with respect to the current value far away from the microtissue (>250 μm, bulk concentration).

To demonstrate the suitability of our platform for SECM-based analysis of microtissues, oxygen measurements are chosen as they are straightforward and direct. Furthermore, the oxygen consumption of microtissues yields essential information on their viability and metabolic activity [9]. The respiratory activity of microtissues is determined by measuring variations in the dissolved oxygen concentration in the solution in the vicinity of the microtissues, and for living microtissues, a gradient in oxygen concentration is expected from the microtissue surface to the bulk of the solution [9], [21]. Therefore, in a first step, the relationship between the oxygen reduction current which is directly proportional to the dissolved oxygen concentration [32], [33], and the distance from the microtissues is determined by scanning over living HeLa microtissues at different heights, along with approach curves over a Petri dish surface and microtissue surface, as shown in Fig. 5B.

As the electrode approaches a Petri dish, the measured current shows a sharp decrease due to hindered diffusion near the surface, within 20 μm from the surface, which correlates well for our electrode dimensions [34]. However, for oxygen consumers such as HeLa microtissues a steady decrease is observed upwards of 250 μm from the surface; this reflects the presence of an oxygen gradient resulting from the active oxygen consumption of the microtissues (See Fig. 5). A large drop (ca. 40%) in oxygen reduction current is observed at the microtissue surface and the concentration progressively increases as the tip moves away from the microtissues, as expected and previously reported in literature [32]. The differences in the approach curves highlight that the drop in reduction current around a tissue is caused by tissue activity and not merely due to hindered diffusion due to the presence of the microtissue.

Subsequently, horizontal line scans are performed along a 1200 μm line above the microtissues, over a microwell (400 μm diameter) and part of the inter-well spacing (400 μm on each side) at a scan rate of 10 μm/s, while ensuring the tip scans across the center of the microtissues (Fig. 5C & D). These scans are repeated at different distances from the tissue surface until no drop in the oxygen reduction current is observed during scanning, i.e. 250 μm from the tissue surface. The horizontal line scans indicate the presence of a hemispherical gradient of oxygen depleted region around the microtissue due to tissue respiration. Interestingly, even at a scanning height of 5 μm from a microtissue, the measured current is not affected by hindered diffusion in the regions between two tissues. This is due to the fact that the HeLa microtissue, 2 days after seeding, protrude >20 μm outside the microwells. Therefore, scanning occurs at >25 μm from the PS surface and at this height bulk concentration of oxygen is measured as shown in Fig. 5B.

### SECM-based live/dead assay

**Figure 6:**
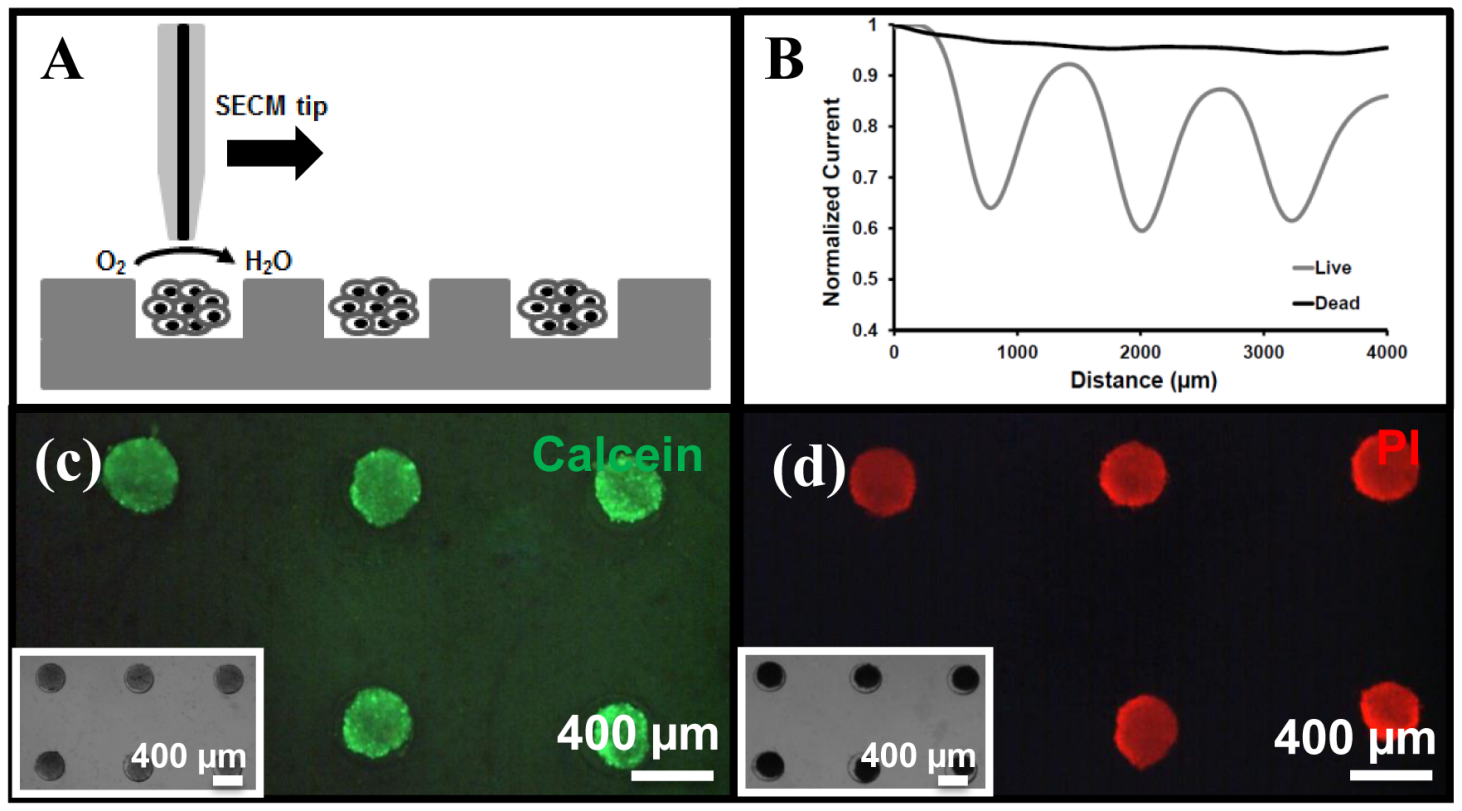
Live/dead assay using SECM and fluorescence microscopy (membrane integrity markers). (A) SECM measurements performed at a constant height of 15 μm above the microtissue surface (scanning in a line across the center of the microtissues). (B) Variations in the oxygen concentration determined as relative variations in the oxygen reduction current for viable tissues (black line) and after exposure to 50% ethanol (grey line). Currents are normalized with respect to the current value far away from the microtissue (>250 μm, bulk concentration). Measurements performed in HEPES buffer at −0.6 V vs. Ag/AgCl with a 10 μm Hg-coated Pt working electrode; scan rate of 10 μm/s. (C &D) Corresponding (fluorescence) microscopy measurements using membrane integrity markers; tissues are initially loaded with calcein (green) (C) which is released out of the cells after ethanol treatment, while PI is taken up (D). Insets in C & D: bright field images of microtissues before and after ethanol treatment.

In a second step, this oxygen measurement approach is applied in a live/dead assay. One day after seeding in the microwell array, HeLa microtissues, which have grown to a size of ca. 370 μm, are exposed to a model drug (50% ethanol) for 45 min, and their viability subsequently analysed using SECM before and after exposure to the drug by monitoring their respiratory activity. First, from the initial characterization of the respiratory activity of live HeLa microtissues, a height of 15 μm with respect to the microtissue surface (i.e., >35 μm from Petri dish surface) is chosen for the live/dead assay, and other measurement conditions are kept the same as before (10 μm/s scanning rate; −0.6 V *vs*. reference Ag wire; ensuring the tip scans above the center of the microtissues). Furthermore, for the live/dead assay, a line scan of 4000 μm is performed over 3 spheroids, as shown on Fig. 6A. As determined earlier, the oxygen reduction current decreases by c.a. 35% over live tissues at that distance (Fig. 6B), while, after ethanol treatment, the oxygen reduction current is unaffected by the presence of tissues (Fig. 6B), as expected. The microtissues do not consume any oxygen since they are dead. It should be noted that we observe a slight drift in the baseline (<5%) while scanning over a series of microtissues primarily due to instability of oxygen reduction [35], [36]. While the faradic currents show improved stability with Hg coating, the instability is not completely eliminated which could be due to incomplete coverage of Hg on Pt surface.

This live/dead assay is conducted in parallel using conventional membrane integrity markers and fluorescence microscopy. Initially, one-day old HeLa microtissues are stained with calcein; they are green, indicating they are viable (Fig. 6C). Exposure to 50% v/v ethanol for 45 min leads to cell death, which is accompanied by damages in the cell membrane, while the overall size of the tissues does not change significantly (Fig. 6C & 6D). As a result, calcein leaks out of the cells, and green fluorescence fades away. Cell death and membrane rupture is confirmed by addition of another membrane-impermeable dye, propidium iodide (PI) to the tissues. Loss of membrane integrity enables the DNA intercalating dye PI to enter the cells, and to subsequently bind to the nucleic acids inside the cells (Fig. 6D). Consequently, dead tissues become PI-positive (red) and calcein-negative, while live tissues remain calcein-positive (green) and PI-negative. These results are in good agreement with the results obtained using SECM analysis of the microtissues and mapping of their respiratory activity.

Microscopy is combined to scanning probe techniques here for a standard live/dead assay on HeLa microtissues. In that context, SECM can yield additional information on biological processes by monitoring local chemical gradients that are otherwise inaccessible using conventional microscopy techniques. Furthermore, and more importantly, this technique is non-invasive, which enables time-lapse monitoring of the tissues. In the future, this combination of the microtissue array in a Petri dish with *in situ* and non-invasive SECM technique will be applied for other cellular assays, *e.g*., to monitor cellular differentiation by continuous monitoring the cell activity over a period of several days.

### Safety Considerations

CAUTION: Mercury ions are highly toxic. Solution must be handled with protective equipment and extreme care to avoid contaminations. All solution and waste from rinsing must be collected and directed to designated heavy metal disposal.

## Supporting Information

File S1 **File S1 contains three supplementary figures (S1, S2 and S3) referenced in the article along with supplementary text.**
(DOCX)

File S2 **File S2 is the supplementary movie referenced in the article.** Time lapse imaging of formation of C2C12 microtissue immediately after seeding. Images are captured every 10 minutes for 24 hours and show the tissue formation process. The tissue formation is spontaneous and the microtissues reach their final diameter ca. 24 h after seeding.
(AVI)

## References

[1] Beaulieu I, Kuss S, Mauzeroll J, Geissler M (2011) Biological Scanning Electrochemical Microscopy and Its Application to Live Cell Studies. Anal Chem 83: 1485–1492. 21214262 10.1021/ac101906a

[2] Sun P, Laforge FO, Mirkin MV (2007) Scanning electrochemical microscopy in the 21st century. Phys Chem Chem Phys 9: 802–823. 17287874 10.1039/b612259k

[3] Roberts WS, Lonsdale DJ, Griffiths J, Higson SP (2007) Advances in the application of scanning electrochemical microscopy to bioanalytical systems. Biosens Bioelectron 23: 301–318. 17869090 10.1016/j.bios.2007.06.020

[4] Stephens DJ, Allan VJ (2003) Light microscopy techniques for live cell imaging. Science 300: 82–86. 12677057 10.1126/science.1082160

[5] Ino K, Nishijo T, Arai T, Kanno Y, Takahashi Y, et al (2012) Local redox-cycling-based electrochemical chip device with deep microwells for evaluation of embryoid bodies. Angew Chem Int Ed Engl 51: 6648–6652. 22639109 10.1002/anie.201201602

[6] Bergner S, Vatsyayan P, Matysik FM (2013) Recent advances in high resolution scanning electrochemical microscopy of living cells-a review. Anal Chim Acta 775: 1–13. 23601970 10.1016/j.aca.2012.12.042

[7] Yasukawa T, Kondo Y, Uchida I, Matsue T (1998) Imaging of cellular activity of single cultured cells by scanning electrochemical microscopy. Chem Lett 8: 767–768.

[8] Shiku H, Shiraishi T, Ohya H, Matsue T, Abe H, et al (2001) Oxygen consumption of single bovine embryos probed by scanning electrochemical microscopy. Anal Chem 73: 3751–3758. 11510844 10.1021/ac010339j

[9] Kaya T, Torisawa YS, Oyamatsu D, Nishizawa M, Matsue T (2003) Monitoring the cellular activity of a cultured single cell by scanning electrochemical microscopy (SECM). A comparison with fluorescence viability monitoring. Biosens Bioelectron 18: 1379–1383. 12896839 10.1016/s0956-5663(03)00083-6

[10] Liu B, Rotenberg SA, Mirkin MV (2000) Scanning electrochemical microscopy of living cells: Different redox activities of nonmetastatic and metastatic human breast cells. P Natl Acad Sci USA 97: 9855–9860. 10.1073/pnas.97.18.9855 PMC27604 10963658

[11] Kuss S, Polcari D, Geissler M, Brassard D, Mauzeroll J (2013) Assessment of multidrug resistance on cell coculture patterns using scanning electrochemical microscopy. P Natl Acad Sci USA 110: 9249–9254. 10.1073/pnas.1214809110 PMC3677433 23686580

[12] Matsumae Y, Arai T, Takahashi Y, Ino K, Shiku H, et al (2013) Evaluation of the differentiation status of single embryonic stem cells using scanning electrochemical microscopy. Chem Commun (Camb) 49: 6498–6500. 23760226 10.1039/c3cc43126f

[13] Obregon R, Horiguchi Y, Arai T, Abe S, Zhou YS, et al (2012) A Pt layer/Pt disk electrode configuration to evaluate respiration and alkaline phosphatase activities of mouse embryoid bodies. Talanta 94: 30–35. 22608410 10.1016/j.talanta.2012.01.059

[14] Pampaloni F, Reynaud EG, Stelzer EHK (2007) The third dimension bridges the gap between cell culture and live tissue. Nat Rev Mol Cell Bio 8: 839–845. 17684528 10.1038/nrm2236

[15] Mehta G, Hsiao AY, Ingram M, Luker GD, Takayama S (2012) Opportunities and challenges for use of tumor spheroids as models to test drug delivery and efficacy. J Control Release 164: 192–204. 22613880 10.1016/j.jconrel.2012.04.045 PMC3436947

[16] Friedrich J, Seidel C, Ebner R, Kunz-Schughart LA (2009) Spheroid-based drug screen: considerations and practical approach. Nat Protoc 4: 309–324. 19214182 10.1038/nprot.2008.226

[17] Rivron NC, Le Gac S, Vrij E, Rouwkema J, Wijnperle D, et al.. (2009) Microfabrication of shaped MM-scale tissues to study vascular development using modular bottom-up approach. MicroTAS 2009: pp. 9–11.

[18] Rivron NC, Vrij EJ, Rouwkema J, Le Gac S, van den Berg A, et al (2012) Tissue deformation spatially modulates VEGF signaling and angiogenesis. P Natl Acad Sci USA 109: 6886–6891. 10.1073/pnas.1201626109 PMC3344996 22511716

[19] Berthier E, Young EW, Beebe D (2012) Engineers are from PDMS-land, Biologists are from Polystyrenia. Lab Chip 12: 1224–1237. 22318426 10.1039/c2lc20982a

[20] Young EWK, Berthier E, Guckenberger DJ, Sackmann E, Lamers C, et al (2011) Rapid Prototyping of Arrayed Microfluidic Systems in Polystyrene for Cell-Based Assays. Anal Chem 83: 1408–1417. 21261280 10.1021/ac102897h PMC3052265

[21] Torisawa YS, Takagi A, Nashimoto Y, Yasukawa T, Shiku H, et al (2007) A multicellular spheroid array to realize spheroid formation, culture, and viability assay on a chip. Biomaterials 28: 559–566. 16989897 10.1016/j.biomaterials.2006.08.054

[22] Shiku H, Goto S, Jung S, Nagamine K, Koide M, et al (2009) Electrochemical characterization of enzymatic activity of yeast cells entrapped in poly(dimethylsiloxane) microwell on the basis of limited diffusion system. Analyst 134: 182–187. 19082191 10.1039/b808428a

[23] Moresco J, Clausen CH, Svendsen W (2010) Improved anti-stiction coating of SU-8 molds. Sens Actuators B Chem 145: 698–701.

[24] Xia YN, Whitesides GM (1998) Soft lithography. Annu Rev Mater Sci 28: 153–184.

[25] Mauzeroll J, Hueske EA, Bard AJ (2003) Scanning electrochemical microscopy. 48. Hg/Pt hemispherical ultramicroelectrodes: Fabrication and characterization. Anal Chem 75: 3880–3889. 14572057 10.1021/ac034088l

[26] Goral VN, Hsieh Y, Petzold ON, Faris RA, Yuen PK (2011) Hot embossing of plastic microfluidic devices using poly(dimethylsiloxane) molds. J Micromech Microeng 21: 017002.

[27] Malek CH, Coudevylle J, Jeannot J, Duffait R (2007) Revisting micro hot-embossing with moulds in non-conventional materials. Microsyst Technol 13: 475–481.

[28] Mehta G, Lee J, Cha W, Tung YC, Linderman JJ, et al (2009) Hard top soft bottom microfluidic devices for cell culture and chemical analysis. Anal Chem 81: 3714–3722. 19382754 10.1021/ac802178u

[29] Tung YC, Hsiao AY, Allen SG, Torisawa YS, Ho M, et al (2011) High-throughput 3D spheroid culture and drug testing using a 384 hanging drop array. Analyst 136: 473–478. 20967331 10.1039/c0an00609b PMC7454010

[30] Wang YL, Balowski J, Phillips C, Phillips R, Sims CE, et al (2011) Benchtop micromolding of polystyrene by soft lithography. Lab Chip 11: 3089–3097. 21811715 10.1039/c1lc20281b PMC3454527

[31] Higuchi A, Aoki N, Yamamoto T, Miyazaki T, Fukushima H, et al (2006) Temperature-induced cell detachment on immobilized pluronic surface. J Biomed Mater Res A 79A: 380–392. 10.1002/jbm.a.30773 16883586

[32] Shiku H, Shiraishi T, Aoyagi S, Utsumi Y, Matsudaira M, et al (2004) Respiration activity of single bovine embryos entrapped in a cone-shaped microwell monitored by scanning electrochemical microscopy. Anal Chim Acta 522: 51–58.

[33] Date Y, Takano S, Shiku H, Ino K, Ito-Sasaki T, et al (2011) Monitoring oxygen consumption of single mouse embryos using an integrated electrochemical microdevice. Biosens Bioelectron 30: 100–106. 21955755 10.1016/j.bios.2011.08.037

[34] Amphleet JL, Denuault G (1998) Scanning electrochemical microscopy (SECM): An investigation of the effects of tip geometry on amperometric tip response. J Phys Chem B 102: 9946–9951.

[35] Li X, Bard AJ (2009) Scanning electrochemical microscopy of HeLa cells – Effects of ferrocene methanol and silver ion. J Electroanal Chem 628: 35–42.

[36] Zhan D, Li X, Nepomnyashchii AB, Alpuche-Aviles MA, Fan FF, et al (2013) Characterization of Ag+ toxicity on living fibroblast cells by the ferrocenemethanol and oxygen response with the scanning electrochemical microscope. J Electroanal Chem 688: 61–68.

