# Supplementary material for: Microstamped Petri Dishes for Scanning Electrochemical Microscopy Analysis of Arrays of Microtissues
Source: PLoS One. 2014 Apr 1;9(4):e93618. doi: 10.1371/journal.pone.0093618 (PMC3972177; doi:10.1371/journal.pone.0093618)
Supplement: File S1 — contains three supplementary figures (S1, S2 and S3) referenced in the article along with supplementary text. (DOCX) [file pone.0093618.s001.docx]

**Supplementary Information**

**Interferometry**

A vertical white light interferometer (Polytec TMS-1200) has been used for surface and depth profiling of patterned Petri dishes. Measurements are performed with a coherent monochromatic light source. In brief, the coherent light is split into two using a beam splitter and a phase difference is introduced between the two waves. This phase difference causes an interference pattern. Physical changes along the vertical axis of the Petri dish introduces further changes in the phase difference which was then used to analyse the surface and depth of the patterned microwell arrays.

**Figure S1.** Homemade setup for hot embossing microwell arrays on commercially available PS Petri dishes. The system consists of a Teflon cup in which the Petri dish is placed Heating is achieved locally in the centre using a brass disc which is feedback-coupled to a soldering station. Pressure is applied using the screw. A PDMS mould is held in the metallic holder and placed in the Petri dish while pressure is applied.

**Figure S2.** (left) Microwell depth for different fabrication parameters (heating and cooling times) measured using white light interferometry (WLI). Although the structures are deeper for shorter heating times (5 min) the deviation in depth is lower for higher heating times (10 min). This is critical as the microwell arrays have to be uniform for homogenous microtissue production. Based on WLI measurements, 180°C with 10 min heating and 15 min cooling have been chosen as optimal fabrication parameters. All Petri dishes used for tissue production were fabricated using the aforementioned settings. (right) 3D reconstruction of a WLI scan showing smooth reformation of the PS surface.

**Figure S3.** Microwell arrays (25 µm diameter and 20 µm depth) stamped in a Petri dish by hot embossing and comprising 100,000 microwells for single cell trapping. This demonstrates the applicability of the technique described in the paper can be scaled up to include a large number of microwells for high throughput testing.
